# Risk factors for neurological complications in severe and rigid spinal deformity correction of 177 cases

**DOI:** 10.1186/s12883-020-02012-8

**Published:** 2020-11-28

**Authors:** Jian Chen, Xie-xiang Shao, Wen-yuan Sui, Jing-fan Yang, Yao-long Deng, Jing Xu, Zi-fang Huang, Jun-lin Yang

**Affiliations:** 1 Spine Surgery Center, Xinhua Hospital Affiliated to Shanghai Jiaotong University School of Medicine, 1665 Kongjiang Road, Shanghai, China; 2 Department of Orthopaedic Surgery, the 1st Affiliated Hospital of Sun Yat-sen University, Guangzhou, Guangdong China

**Keywords:** Severe spinal deformity, Neurological complication, Osteotomy, Risk factor

## Abstract

**Background:**

Difficult procedures of severe rigid spinal deformity increase the risk of intraoperative neurological injury. Here, we aimed to investigate the preoperative and intraoperative risk factors for postoperative neurological complications when treating severe rigid spinal deformity.

**Methods:**

One hundred seventy-seven consecutive patients who underwent severe rigid spinal deformity correction were assigned into 2 groups: the neurological complication (NC, 22 cases) group or non-NC group (155 cases). The baseline demographics, preoperative spinal cord functional classification, radiographic parameters (curve type, curve magnitude, and coronal/sagittal/total deformity angular ratio [C/S/T-DAR]), and surgical variables (correction rate, osteotomy type, location, shortening distance of the osteotomy gap, and anterior column support) were analyzed to determine the risk factors for postoperative neurological complications.

**Results:**

Fifty-eight patients (32.8%) had intraoperative evoked potentials (EP) events. Twenty-two cases (12.4%) developed postoperative neurological complications. Age and etiology were closely related to postoperative neurological complications. The spinal cord functional classification analysis showed a lower proportion of type A, and a higher proportion of type C in the NC group. The NC group had a larger preoperative scoliosis angle, kyphosis angle, S-DAR, T-DAR, and kyphosis correction rate than the non-NC group. The results showed that the NC group tended to undergo high-grade osteotomy. No significant differences were observed in shortening distance or anterior column support of the osteotomy area between the two groups.

**Conclusions:**

Postoperative neurological complications were closely related to preoperative age, etiology, severity of deformity, angulation rate, spinal cord function classification, intraoperative osteotomy site, osteotomy type, and kyphosis correction rate. Identification of these risk factors and relative development of surgical techniques will help to minimize neural injuries and manage postoperative neurological complications.

## Background

Severe spinal deformities are characterized by complexity of the anatomical structure and rigidity, and are often accompanied by severe cardiopulmonary and gastrointestinal dysfunction, which can lead to physical disability or even death. Spine osteotomy has rapidly developed and is widely used to treat severe scoliosis, with good outcomes. However, difficult screw placement, the complexity of the osteotomy and orthopedic procedures, significantly increase the risk of intraoperative neurological injury. Previous works reported that the incidence of postoperative neurological complications in spinal deformity is 4.0% ~ 21.2% [1–4]. Lenke et al. reported an incidence of intraoperative neurophysiological monitoring events of 20% for patients undergoing vertebral column resection (VCR), while the incidence of transient neurological damage after the operation was 5.7% [3]. Kelly et al. reported an incidence of neurological complications after three-column osteotomy of 9.9% [4]. Neurological complications of spinal surgery result from several surgical factors, including compression and traction of neural structures, osteotomy procedure-related injury, hypotension, and spinal cord ischemia [5–7].

However, few works have comprehensively clarified the potential risk factors for postoperative neurological complications in patients who have undergone severe spinal deformity surgery. This study retrospectively analyzed the clinical, imaging and intraoperative neuromonitoring data of 177 patients with a severe spinal deformity. The preoperative, intraoperative, and postoperative potential risks factors for neurological complications were analyzed and compared between neurological complication (NC) and non-NC groups.

## Methods

The data of 177 consecutive patients (84 males and 93 females; mean age, 21.0 years [range: 8–56 years]) who underwent severe rigid spinal deformity osteotomy correction between September 2006 and June 2017 were analyzed retrospectively. The inclusion criteria were: 1) scoliosis curve > 80° or kyphosis > 80°; 2) complete medical records, containing general, imaging, intraoperative neurological monitoring, surgical procedure, and postoperative complication data; 3) normal neurological function before surgery; and 4) a minimum follow-up of 2 years. As shown in Table 1, the etiological diagnoses of the cases were idiopathic scoliosis (78 cases), congenital scoliosis (29 cases), neuromuscular scoliosis (32 cases), neurofibromatosis (17 cases), tuberculosis (9 cases), revision (8 cases), and other (4 cases) (Table 1). The types of curvature included scoliosis (25 cases), kyphosis (11 cases), and kyphoscoliosis (141 cases). According to our previously proposed spinal cord functional classification system (Table 2) [1], there were 108 type A, 51 type B, and 18 type C cases. Preoperative halo gravity traction was provided to patients who had a baseline FVC <40%, and these patients did not undergo surgery until they had an FVC >40%. Fifty-five patients (31.1%) underwent gravity cranial ring traction. The types of osteotomy included Smith–Petersen osteotomy (SPO, 39 cases), pedicle subtraction osteotomy (PSO, 29 cases), bone-disc-bone osteotomy (BOBO, 43 cases), posterior VCR (53 cases), and multi-segmental VCR (35 cases). All pedicle screws were inserted manually.

**Table 1:** The demographics, spinal cord function classification, and surgical parameters of patients

Characteristic	Mean ± SEM
Age	21.0 (8–56)
Sex(M/F)	84/93
Diagnosis	IS(78),CS(29),NM(32),NF(17), TB(9),revision(8), others(4)
Curve type	
Scoliosis	25
Kyphosis	11
Kyphoscoliosis	141
Spinal cord function classification	
Type A	108
Type B	51
Type C	18
Halo gravity traction	55/177
Osteotomy	
SPO	39
PSO	29
PDO	43
VCR	53
MVCR	35

**Table 2:** Yang’s Classification System of Severe Spinal Deformities

Classification	Neurological Symptoms	MRI Findings	Evoked Potentials
Type A	–	–	–
Type B	–	-or+	-or+
Type C	+	-or+	+

*MRI* magnetic resonance imaging; No, neurological symptoms not present, no spinal cord malformations found on MRI, no positive change in evoked potentials; Yes, neurological symptoms present, spinal cord malformations found on MRI, positive change in evoked potentials

Multimodal intraoperative neuromonitoring evoked potentials (somatosensory evoked potentials [SSEP]/ transcranial motor evoked potentials [MEP]/ descending neurogenic evoked potentials [DNEP]) were applied as described previously [1]. In this study, the alarm criteria of EP were as follows: 1) MEP: amplitude disappeared in unilateral or bilateral lower extremities and not restored within 10 min [8]; 2) SSEP: the amplitude decreased by > 50% and/or the latency prolonged by > 10% compared with baseline level [9]; 3) DNEP: the amplitude reduced by > 80% and/or the latency prolonged by > 10% compared with baseline level [10]. The specific procedures were as follows when EP events happens: 1. To stop the operation intraoperatively and check the current anesthesia and physiological states including anesthetic potency, muscle relaxants, inhalation gas and blood pressure; and properly increase the blood pressure and reduce the anesthesia depth. 3. The pedicle screw should be removed immediately when EP events happened in screws insertion. The operation should be stopped and correction can be reduced, and the condition of the spinal cord should be checked when EP events happened during the osteotomy and correction. 4.Routine wake-up test was performed immediately after surgery. The false positive event criteria were as follows: 1. there was no correlation of intraoperative evoked potentials events and surgical procedures, with recovery only after observation or routine treatment; 2. No recovery or incomplete recovery of MEP amplitude after routine treatment when there was no corresponding postoperative neurological injury. Neurological deficit were determined by a physician according to the Frankel grading method [11].

All cases were categorized into the NC or non-NC group, and the general data of the two groups were compared. The baseline demographics, preoperative interventions, surgical variables, radiographic parameters (including curve type, curve magnitude [coronal and sagittal Cobb angles], and coronal/sagittal/total deformity angular ratios [C/S/T-DAR, T-DAR = C-DAR + S-DAR]) were measured in both groups to determine the possible risk factors for postoperative neurological complications. The scoliosis and kyphosis correction rate were determined as follows: (preoperative Cobb’s angle − post-operative Cobb’s angle)/pre-operative Cobb’s angle. All radiographic parameters were evaluated by the same physician (Z.H.).

### Statistical analysis

Pearson’s chi-square test was performed to analyze the categorical variables, and the independent samples *t*-test and analysis of variance were used for analyzing continuous variables. All analyses were performed using SPSS 19.0 software (SPSS Inc., Chicago, IL, USA). A *P*-value < 0.05 was considered significant.

## Results

### Baseline measurements

**Fig. 1:**
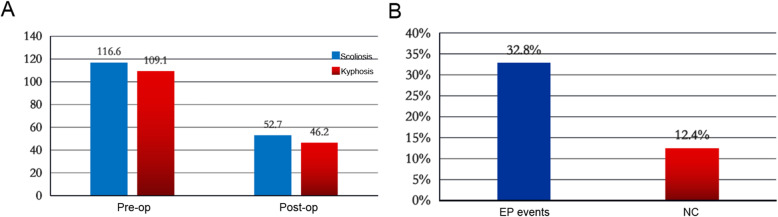
Data of spinal deformity correction and neurological complications incidence in all patients **a** The patient’s preoperative and postoperative scoliosis angle and kyphosis angle. **b** The incidence of evoked potential events and neurological complications

A total of 177 cases were included in this study. The preoperative scoliosis Cobb angle was 116.6 ± 21.2° and the scoliosis correction rate was 54.8%. The kyphosis Cobb angle was 109.1 ± 23.3° and the correction rate was 57.9% (Fig. 1a). In total, 58 of 177 patients (32.8%) had EP events during the operation. Twenty-two patients (12.4%) developed neurological complications after surgery (Fig. 1b). All patients with postoperative neurological impairment have intraoperative EP alarm events. The positive predictive value and negative predictive value of EP events were 82.6 and 100%, respectively. Twenty-two patients of 58 patients (37.9%) patients with EP positive events experienced postoperative neurological impairment (Table 3).

**Table 3:** Data of Intraoperative Outcomes in NC group

Case	Preoperative traction	Osteotomy grade	Blood loss (mL)	Operative time (min)	The stage of EP events	Postoperative Spinal Cord Function	Complication
1	Yes	2	1000	600	Correction	Frankel grade C	Pulmonary infection
2	No	3	2000	370	Osteotomy	Frankel grade D	–
3	No	4	1550	420	Screw insertion and osteotomy	Frankel grade D	–
4	No	4	8200	550	Correction	Frankel grade A	–
5	Yes	5	5900	1100	Osteotomy and correction	Frankel grade C	–
6	Yes	5	3600	445	Osteotomy	Frankel grade D	–
7	No	3	3200	510	Osteotomy	Frankel grade D	Wound infection and urinary tract infection
8	Yes	5	3700	600	Correction	Frankel grade C	–
9	Yes	5	5968	975	Osteotomy and correction	Frankel grade B	–
10	No	5	4999	665	Correction	Frankel grade C	–
11	Yes	5	4050	580	Osteotomy and correction	Frankel grade C	–
12	Yes	6	1200	420	Osteotomy	Frankel grade D	Wound infection
13	Yes	6	1200	390	Osteotomy	Frankel grade D	Wound infection
14	Yes	6	960	353	Screw insertion and osteotomy	Frankel grade D	–
15	Yes	6	2400	560	Correction	Frankel grade D	Wound infection
16	No	4	1100	440	Osteotomy	Frankel grade C	Hemopneumothorax
17	Yes	5	3800	590	Osteotomy and correction	Frankel grade B	Hemorrhage
18	No	6	2500	550	Correction	Frankel grade D	–
19	Yes	6	5779	590	Osteotomy and correction	Frankel grade C	Cerebrospinal fluid leakage
20	Yes	5	3300	615	Correction	Frankel grade D	–
21	Yes	6	4600	550	Osteotomy	Frankel grade C	–
22	No	6	1500	550	Correction	Frankel grade D	–

### Preoperative risk factors

No significant difference in gender or curvature type was observed between the two groups (both, *P* > 0.05, Table 4). However, our results suggest that patients in the NC group were older than those in the non-NC group. Postoperative neurological complications were related to the etiology of spinal deformity (*P* = 0.001, Table 4) and the spinal cord functional classification (*P* < 0.001, Table 4). More non-adolescent idiopathic scoliosis (AIS) patients were in the NC group than in the non-NC group. According to the spinal cord functional classification we proposed previously [1], a lower proportion of type A, and a higher proportion of type C, cases were observed in the NC group. We also divided the patients into three groups according to the functional classification of severe spinal deformity. The incidence rates of EPs and neurological complications in the type B and type C groups were significantly higher than those in the type A group, suggesting that preoperative spinal cord function was significantly related to postoperative neurological complications (*P* < 0.001, Table 5). Furthermore, the NC group had a larger preoperative angle of scoliosis (*P* = 0.017, Table 4) and preoperative angle of kyphosis (*P* = 0.001, Table 4) than the non-NC group. The NC group had a bigger S-DAR (*P* = 0.001, Table 4) and T-DAR (*P* = 0.001, Table 4) than the non-NC group, while there were no significant differences in flexibility of the main curve and C-DAR preoperatively (*P* > 0.05, Table 4). More patients in the NC group needed halo-gravity traction (*P* < 0.001, Table 4).

**Table 4:** Related factors of neurogical complications between NC group and non-NC group

Factors	NC group(*n* = 22)	Non-NC group(*n* = 155)	*P* value
Age	24.6 ± 8.7	20.5 ± 8.2	0.032
Sex(M/F)	12/10	72/83	0.459
Diagnosis	IS(7),CS(5),NM(6), TB(2),revision(2)	IS(71),CS(24),NM(26),NF(17), TB(7),revision(6),other(4)	0.032
Osteotomy			
SPO	1	38	0.001
PSO	2	27	
PDO	3	40	
VCR	8	45	
MVCR	8	27	
Curve type			
Scoliosis	0	25	0.122
Kyphosis	2	9	
Kyphoscoliosis	20	121	
Spinal cord function			
Type A	6	102	< 0.001
Type B	7	44	
Type C	9	9	
Angle of scoliosis (°)	129.6 ± 26.2	114.9 ± 25.6	0.017
Scoliosis correction rate (%)	50.5% ± 18.8%	55.4% ± 12.4%	0.123
Angle of kyphosis (°)	130.8 ± 31.3	105.5 ± 33.2	0.001
Kyphosis correction rate (%)	49.8% ± 20.4%	59.3% ± 14.3%	0.008
C-DAR	19.3 ± 6.6	18.1 ± 5.5	0.344
S-DAR	23.2 ± 7.9	18.1 ± 8.1	0.007
T-DAR	40.8 ± 12.6	32.4 ± 13.2	0.006
Flexibility (%)	14.4 ± 11.5	15.6 ± 10.9	0.672
Halo gravity traction	14/22	41/113	< 0.001
Osteotomy site			
HT	2	6	0.037
MT	10	58	
LT	10	72	
L	0	19	
Anterior strut graft	7/22	27/155	0.115
Shortening distance of osteotomy gap (cm)	3.8 ± 0.8	3.5 ± 0.8	0.159
Blood loss	2605.7 ± 131.3	3295.7 ± 425.3	0.073
Operative time	472.1 ± 8.3	564.5 ± 37.8	0.001

**Table 5:** The incidence of evoked potentials and neurological complication for different types of spinal cord function classification

Classification	Incidence of EP events	Incidence of NC events
Type A	26.90%	5.60%
Type B	39.20%	13.70%
Type C	50%	50%
*p*	0.078	< 0.001

### Intraoperative risk factors

There is no statistically different in the scoliosis correction rate between the two groups (*P* > 0.05, Table 4). However, the NC group had a larger kyphosis correction rate (*P* = 0.008, Table 4) than the non-NC group. Furthermore, postoperative neurological complications were related to the osteotomy method (*P* = 0.001, Table 4). The NC group tended to undergo high-grade osteotomy: osteotomy of grade 5 or above accounted for 72.8% of all cases in the NC group, while it only accounted for 46.4% of those in the non-NC group. The osteotomy segment (*P* = 0.037, Table 4) was more likely to be the upper/middle thoracic vertebrae in the NC group than the non-NC group. In addition, patients in the NC group had a longer shortening distance of the osteotomy gap, a higher rate of anterior column support placement in the osteotomy area and more blood loss during the operation, although the differences were not significant (*P* > 0.05, Table 4). Furthermore, the operation time of NC group was longer than the non-NC group (*P* = 0.001, Table 4).

## Discussion

Osteotomy for severe rigid spinal deformity is a technically demanding and high-risk procedure. The orthopedic outcomes of severe spinal deformity operations have significantly improved in the past few decades with advancements in the osteotomy technique. However, difficult screw placement, the complex nature of osteotomy and orthopedic procedures, significantly increase the risk of postoperative neurological complications. Suk et al. reported that the incidence of postoperative complications was 40.3% in 233 cases of severe spinal deformity who underwent posterior vertebral column resection (PVCR); 21.2% of patients had a transient neurological injury, and 4.5% had a permanent postoperative neurological injury [12]. Lenke et al. reported that the incidence of EP events was 27.0% in 147 cases of severe spinal deformity, while the incidence of neurological complications was 6.1% [13]. Several surgical factors are involved in the neurological complications that may arise from spinal surgery, including compression and traction of neural structures, osteotomy related injuries, hypotension, and spinal cord ischemia [5–7, 14, 15]. In total, 58 of 177 patients (32.8%) had EP events during the operation. Twenty-two patients (12.4%) developed neurological complications after surgery. All patients with postoperative neurological impairment have intraoperative EP alarm events. Twenty-two patients of 58 patients (37.9%) patients with EP positive events experienced postoperative neurological impairment. Our results are consistent with previous literature. However, the potential risk factors for neurological complications due to a severe thoracic deformity have not been clearly clarified. To help the surgeon evaluate preoperative and intraoperative surgical risk factors and reduce postoperative neurological complications, this study examined the potential risk factors for neurological complication (pre-, intra- and postoperative factors) in patients with severe rigid spinal deformity.

Age, etiology, severity of the spinal deformity, deformity angular ratio, and spinal cord functional classification were significantly correlated with the incidence of postoperative neurological complications. Our results suggest that age was closely related to neurological complications, which may be due to senescence-induced long-term stress asymmetry of the spine, proliferation and ossification of small joints, reduced flexibility of spinal muscles and ligaments, and a decline the tolerance and repair ability of the spinal cord [16–19]. We have proposed a spinal cord functional classification to stratify patients undergoing severe spinal deformity correction based on the risk of neurological injury [1]. We found that the incidence rates of EP events and neurological complications in the type B and type C groups were significantly higher than those in the type A group, suggesting that patients with preoperative clinical symptoms and radiographic and EP abnormalities are highly amenable to mechanical stimulation. Preoperative radiological parameters, including the preoperative scoliosis/kyphosis angle and the spinal coronal/sagittal deformity angular ratio (S-DAR and T-DAR), were significantly different between the NC and non-NC groups. Similar to results reported in the literature, a greater sagittal deformity angular ratio was associated with a higher risk of neurological injury. When S-DAR > 15°, the incidence of neurological complications (12.5% vs. 0%) and spinal cord monitoring events (34% vs. 15.1%) are greater than when S-DAR < 15° [3]. Another study demonstrated that when T-DAR > 45° and S-DAR > 22°, the “MEP alarm” rate was 75%. When S-DAR > 28°, the MEP alarm rate was 90% [20]. Severe spinal deformities require a higher osteotomy level. Our previous study also showed that the preoperative maximum kyphosis and S-DAR may affect the surgeon’s decision regarding the osteotomy grade [21]. Kyphosis can easily cause a spinal cord injury during the operation, because: 1) the anterior spinal artery is easily affected; 2) the anterior horn of the spinal cord is easily compressed; 3) a high-grade osteotomy requires more anterior column manipulation; and 4) the anterior horn of the spinal cord is easily pulled and injured during the correction.

Intraoperative risk factors including the osteotomy site, osteotomy type, shortening distance of the osteotomy gap, and correction rate of the deformity angle are closely related to the incidence of postoperative neurological complications. Our results showed that the NC group tended to receive high-grade osteotomy; osteotomy of grade 5 or above accounted for 72.8% of all osteotomies in the NC group, but only 46.4% in the non-NC group. Osteotomies of grade 5 or above carry a risk of neurological injury risk for the following reasons. First, high-grade osteotomy on the anterior and middle column is complicated and the spinal cord is more easily pulled out of position; second, the blood supply to the spinal cord is more compromised (including venous plexus hemorrhage and ligation of vertebral segment blood vessels); third, the blood supply to the spinal cord is reduced due to excessive bleeding during the operation; and fourth, the spinal cord can kink, which affects the sagittal/coronal plane when the osteotomy area is closed. Our results suggest that osteotomy at higher thoracic levels poses higher risk of MEP alarm and postoperative neurological deficits. The cross-section of higher segments of the spinal canal is smaller than that of lower segments, and osteotomy can easily lead to neurological complications [22]. The blood supply to the upper spinal cord in the transitional zone of two different vascular distribution areas is called the dangerous zone. If one or more related intercostal arteries are injured or ligated, the thoracic spinal cord may not receive a sufficient blood supply. Thus, the surgeon must be vigilant during the surgical procedure to reduce the risk of neurological deficit during a thoracic operation and raise blood pressure properly in time when evoked potential changes. In the present study, no difference was detected in the shortening distance of the osteotomy gap between the two groups, nor in the rate of use of anterior column support. In our previous study, the convex side of the vertebral body was longer than the central part of the vertebral canal (33.1 vs. 20.1 mm), according to pre- and postoperative 3D reconstructed computed tomography (CT) scans [23]. Therefore, the shortening distance of the convex vertebral body was greater than that of the actual spinal cord. In our previous study, the orthopedic outcomes and incidence of neurological complications were similar between PVCR without anterior support (*n* = 36) and with anterior support (*n* = 21) groups; moreover, the failure rate of internal fixation was lower in the PVCR without anterior support group [24], suggesting that PVCR without anterior support, with a mean shortening of the spinal column of 3.7 cm, is safe provided that postoperative fusion is ensured. Spinal column shortening through bone-on-bone fusion is advisable in appropriately selected patients, as the central vertebral canal is markedly shorter than the convex vertebral body [25]. Preoperative sagittal kyphosis and S-DAR are important factors associated with postoperative neurological complications, as described above. A statistical difference in the intraoperative kyphosis correction rate was observed between the two groups; the correction rate, and especially the kyphosis correction rate, should be reduced for patients at high risk of spinal cord injury.

Postoperative hematoma is an important factor contributing to postoperative neurological complications, and is often influenced by factors such as incomplete hemostasis, excessive intraoperative trauma, and coagulation dysfunction [26–28]. If postoperative muscle strength continues to decline and a postoperative CT or magnetic resonance image shows a compressed hematoma, surgery should be performed immediately. However, spinal cord ischemia or excessive correction may result in delayed spinal cord injury and a decline in muscle strength, although some patients can recover with loosening of the fixed bar.

## Conclusions

Severe rigid spinal deformity osteotomy correction carries a high risk of postoperative neurological complications. The present study found that neurological complications were closely related to preoperative age, etiology, spinal cord functional classification, severity of deformity and S-DAR, intraoperative kyphosis correction rate, osteotomy site, and osteotomy grade. The identification and evaluation of risk factors for neurological complications, and the development of surgical techniques help the surgeon to manage these complications and minimize neural injuries.

## Data Availability

The datasets used and/or analyzed during the current study are available from the corresponding author on reasonable request.

## Abbreviations

C/S/T-DAR: Coronal/sagittal/total deformity angular ratio
NC: Neurological complication
SPO: Smith–Petersen osteotomy
PSO: Pedicle subtraction osteotomy
BOBO: Bone-disc-bone osteotomy
VCR: Vertebral column resection
SSEP: Somatosensory evoked potentials
DNEP: Descending neurogenic evoked potentials
MEP: Transcranial motor evoked potentials
AIS: Adolescent idiopathic scoliosis
CT: Computed tomography
EP: Evoked potential
